# Leveraging Electronic Health Record Data From a Novel Standardized Note Template to Study Elder Abuse in Veterans

**DOI:** 10.1093/geroni/igaf122.4396

**Published:** 2025-12-31

**Authors:** Lihan Kao, Maria Mor, Hongwei Zhang, Tony Rosen, Lena Makaroun

**Affiliations:** University of Pittsburgh School of Medicine, Pittsburgh, Pennsylvania, United States; VA Pittsburgh Healthcare System, Pittsburgh, Pennsylvania, United States; VA Pittsburgh Healthcare System, Pittsburgh, Pennsylvania, United States; Weill Cornell Medicine / NewYork-Presbyterian Hospital, New York, New York, United States; VA Pittsburgh Healthcare System/University of Pittsburgh, VA Pittsburgh Healthcare System, Pennsylvania, United States

## Abstract

Though healthcare systems play an important role in identifying and responding to elder abuse (EA), inadequate electronic health record (EHR) data have hindered EA surveillance and research efforts. In 2025, the Veterans Health Administration (VHA) implemented a national standardized EHR note template to document reports of suspected abuse/neglect. In this retrospective descriptive study, we examined VHA EHR data from the 10-site pilot and initial four months of national implementation of the new note template (3/2024 – 6/2025). To focus on EA, we restricted analyses to veterans aged ≥60 years and excluded reports to Child Protective Services. The final analytic sample consisted of 1,982 reports for 1,684 unique veterans. Reported veterans had a median age of 76 years and were 92.8% male, 20.2% non-Hispanic Black, 3.9% Hispanic, and 30.3% married. While most (86.4%) had one report filed over the study period, 11.0% had two, and 2.7% had ≥3. EA occurred most often in the veteran’s home (78.5%). The most frequent abuse types were self-neglect (36.3%), multiple abuse types (24.2%), and financial exploitation (17.6%). Among poly-victimization reports, the most frequent combination was financial exploitation and self-neglect. Among non-self-neglect cases (N = 1,253), the most commonly reported suspected harmers were multiple individuals (23.7%) or adult children (19.2%). This first system-wide description of EA in a national healthcare system, enabled by standardized documentation, highlights prevalent self-neglect, poly-victimization, and multiple suspected harmers, underscoring the complexity of EA identified in healthcare settings. Future efforts using data from structured EHR tools may improve EA detection and prevention efforts.

